# MI-181 enhances ciliation and cilia length in a cigarette smoke exposed airway epithelial model

**DOI:** 10.1038/s41598-026-37296-2

**Published:** 2026-01-24

**Authors:** Ankur A. Gholkar, Caroline Cherry, Thomas V. Gimeno, Claire Nocon, Chunni Zhu, Brigitte N. Gomperts, Jorge Z. Torres

**Affiliations:** 1 UCLA Department of Chemistry and Biochemistry, Los Angeles, CA 90095 USA; 2 Department of Pediatrics, UCLA Children’s Discovery and Innovation Institute, Mattel Children’s Hospital, David Geffen School of Medicine, Los Angeles, CA 90095 USA; 3 Environmental and Molecular Toxicology Interdepartmental Program, Los Angeles, CA 90095 USA; 4 Department of Neurology, David Geffen School of Medicine, Los Angeles, CA 90095 USA; 5 Jonsson Comprehensive Cancer Center, Los Angeles, CA 90095 USA; 6 Eli and Edythe Broad Stem Cell Research Center, Los Angeles, CA 90095 USA; 7 Division of Pulmonary and Critical Care Medicine, Department of Medicine, David Geffen School of Medicine, Los Angeles, CA USA; 8 Molecular Biology Institute, University of California, Los Angeles, CA 90095 USA; 9 UCLA Department of Chemistry and Biochemistry, Los Angeles, CA 90095 USA

**Keywords:** Cell biology, Diseases, Medical research

## Abstract

Multiciliated airway epithelial cells possess motile cilia, which are essential for mucociliary clearance, facilitating the removal of particulates from the respiratory system. Previous studies showed that exposure to cigarette toxins causes damage to motile cilia formation, length, and function, and can lead to reduced mucociliary clearance and lung diseases like COPD. Given the limited options for treating smoking-related diseases, it is imperative to define novel therapeutics to address this need. Recently, we discovered that, contrary to its ability to depolymerize microtubules, the small molecule MI-181 can induce ciliogenesis and increase the length of primary cilia in retinal pigment epithelial cells without adverse effects on cell health. Here, we utilized a human airway basal stem cell derived air-liquid interface mucociliary airway epithelium model system, coupled with smoke exposure, to test the effect of MI-181 on motile cilia. We determined that MI-181 promotes the recovery of motile cilia length. Additionally, the effect of MI-181 on the area covered by motile cilia and levels of the FOXJ1 motile cilia transcription factor showed inter-donor heterogeneity. Importantly, transmission electron microscopy analysis of motile cilia axonemes showed that MI-181-treated motile cilia displayed a normal 9 + 2 arrangement of microtubules. Together, these data suggest that MI-181 promotes the recovery of motile cilia length after smoke exposure and that these cilia are structurally intact.

**Supplementary Information:**

The online version contains supplementary material available at 10.1038/s41598-026-37296-2.

## Introduction

Within the airway epithelial cell layer, there are two types of cilia: primary cilia, which are found in a variety of cell types, and motile cilia, which are found in differentiated multiciliated cells^1^. Similar to primary cilia, motile cilia are microtubule-based structures that are built from the mother centriole and protrude from the cell^2^. However, the axoneme has a more complex structure with a central pair of microtubules and additional protein factors that stabilize the structure and function in motility, like the dynein arms^2,3^. Multiciliated cells play a crucial role in mucociliary clearance, working in tandem with mucus-producing goblet cells to trap particles in the mucus and propel them via unidirectional ciliary rotation (cilia beating) towards the throat, thereby removing particulates from the respiratory system^2,4^. Dysregulation of motile cilia formation, length, and function in airway multiciliated cells can lead to lung disease. For example, the loss of ciliated cells is well-documented in chronic obstructive pulmonary disease (COPD)^5^. Additionally, over the past decades, numerous studies have shown that exposure to cigarette and e-cigarette toxins damages ciliated airway epithelial cells, where the number of ciliated cells and cilia length decrease^2^^5–8^^9^.

Motile cilia homeostasis relies on numerous protein factors and signaling pathways that can be deregulated by smoke toxins. In particular, soluble tubulin monomers and the intraflagellar transport (IFT) machinery are critical for cilia formation, maintenance, and function^10^. Interestingly, Hessel et al.^11^ showed that the gene expression of eight IFT components (KIF3A, IFT172, IFT54, IFT57, CLAUP1, DYNCH21, IFT144, and IFT43) was downregulated in airway epithelial cells from smokers and smokers with COPD compared to nonsmokers. While Brekman et al.^12^ determined that cigarette smoke extracts suppressed the expression of IFT components (KIF21A, DYNC2H1, IFT57, IFT172, TTC26, BBS5, and CLUAP1) in airway epithelial cells through the downregulation of the FOXJ1 transcription factor. Both studies demonstrated that the downregulation of IFT components was correlated with a decrease in motile cilia formation and a shortening of motile cilia. Notably, a study aimed at discovering Dynein inhibitors identified the compound Ciliobrevin D, which inhibited Dynein-2 function, a crucial component of the IFT system, leading to the suppression of ciliogenesis and cilia length in cells with primary cilia^13^. Our previous study demonstrated that the microtubule depolymerizing compound MI-181^14,15^ could induce ciliogenesis and increase primary cilia length in retinal pigment epithelial cells that had been treated with the IFT inhibitor Ciliobrevin D and either lacked cilia or had compromised, shortened cilia^16^. However, whether MI-181 could induce motile cilia formation and restore motile cilia length in cigarette smoke-exposed airway epithelium remained to be tested.

In vivo and in vitro, motile cilia gradually appear during airway epithelial cell differentiation to form a stratified airway epithelium^2^. This has allowed researchers to study ciliated airway epithelial cells in vitro. In this system, human airway basal stem cells (ABSCs) are differentiated using an air-liquid interface (ALI) procedure to generate a pseudostratified epithelium with multiciliated cells, where motile cilia protrude from the apical side of the cells and are exposed to air, thereby mimicking the in vivo environment^5,17,18^. These airway epithelium systems can be treated with chemicals or particulates to assess their effect on the health of the airway epithelium, and more specifically, to measure their impact on the percentage of ciliated cells, cilia length, and cilia motility. For example, Aufderheide et al.^18^ showed that ALI-derived human bronchial epithelial cells (HBECs) exposed to cigarette smoke lost ciliated cells or had ciliated cells with shortened cilia. Similarly, Gohy et al.^5^ analyzed ALI-derived HBECs from control non-diseased patients and COPD patients and found that the epithelium of COPD patients had fewer ciliated cells and/or shorter cilia than that of the control patients. Additionally, Durra et al.^17^ demonstrated that ABSC exposure to e-cigarette toxins induced negative changes in the structure and function of airway motile cilia. Furthermore, this system is also amenable to the discovery and testing of novel agents that can restore the percentage of multiciliated cells, cilia length, and cilia motility in the airway epithelium after it has been damaged by toxins from cigarettes and e-cigarettes.

Given the limited options for treating COPD and other smoking-related lung diseases, it is imperative to define novel treatments that can improve health outcomes in these patients. Here, we have used an ABSC derived ALI airway epithelium system to test the ability of the small molecule MI-181 (which we previously showed restores primary cilia biogenesis and cilia length to epithelial cells that otherwise lacked cilia or had shortened defective cilia) to restore motile cilia biogenesis, length, and structure in airway epithelium that has been exposed to cigarette smoke.

## Results

### MI-181 enhances recovery of motile cilia length and ciliated cell density in cigarette smoke-treated ABSC ALI cultures

**Fig. 1:**
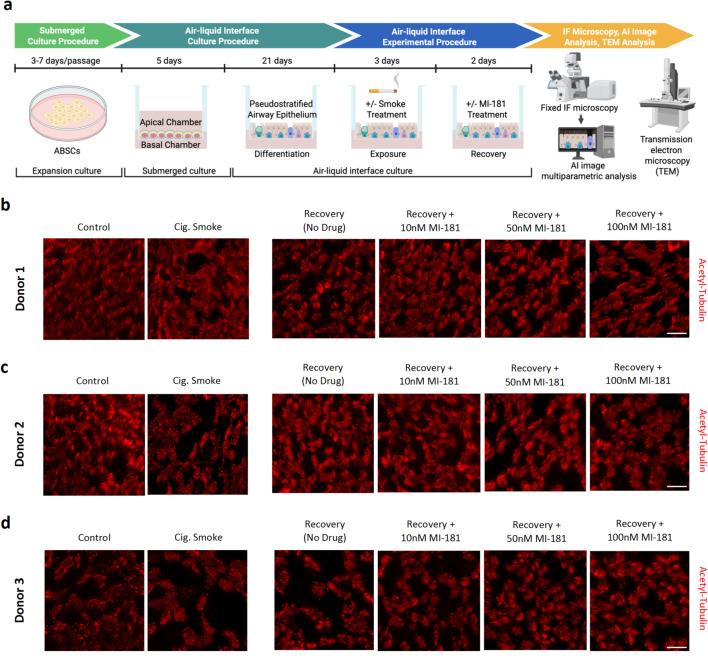
Establishment of an airway basal stem cell (ABSC) air-liquid interphase (ALI) experimental system used to test the effect of MI-181 on motile cilia in multiciliated cells. (**a**) Schematic of experimental approach used to establish donor-derived ABSC ALI cultures; treatment with cigarette smoke; recovery in the presence or absence of MI-181; immunofluorescence microscopy (IF) imaging and artificial intelligence image-based analysis of cilia length, area covered by cilia, and FOXJ1 levels in multiciliated cells; and transmission electron microscopy to assess motile cilia axoneme structure. (**b-d**) IF microscopy of ABSC ALI cultures derived from three separate donors. ABSC ALI cultures were treated with or without cigarette smoke and allowed to recover in the absence or presence of increasing concentrations of MI-181 (10nM, 50nM, and 100nM). Two days post-recovery, ABSC ALI cultures were fixed and stained for cilia axonemes using an anti-acetylated tubulin antibody (red). Scale bars indicate 20 μm.

Patients who smoke downregulate components of the IFT machinery, which is crucial for building motile cilia, thereby inhibiting cilia production and/or producing shortened defective motile cilia^11^. Our previous study with MI-181 demonstrated that it could restore primary cilia and cilia length in cells treated with an IFT inhibitor^16^. Therefore, we sought to test the ability of MI-181 to restore ciliation and cilia length in the airway epithelium, which normally maintains a population of ciliated cells but lacks ciliated cells or has cells with compromised, shortened cilia due to exposure to cigarette smoke. To do this, we obtained human ABSCs from the tracheal and bronchial epithelium of three donors with no history of smoking or lung disease. These primary ABSCs were expanded in submerged culture using PneumaCult-EX Plus medium, transferred to transwell chambers containing porous membrane inserts, and grown to confluency over five days (Fig. 1a). Next, the ABSCs were subjected to an air-liquid interface (ALI) culture procedure^17^, in which the medium was removed and PneumaCult-ALI medium was added only to the lower basal chamber. The cells were allowed to differentiate into a pseudostratified airway epithelium for 21 days (Fig. 1a). The ABSC ALI cultures, containing ciliated cells, were then subjected to either cigarette smoke (using research-grade cigarettes 1R6F) or a control condition (no smoke exposure) for three consecutive days, with one three-minute exposure per day, in a smoke chamber (Fig. 1a)^19^. ABSC ALI cultures were then allowed to recover in the presence or absence of varying concentrations of MI-181 (10nM, 50nM, and 100nM) for two days (Fig. 1a). ABSC ALI cultures were then fixed with paraformaldehyde (PFA) and stained for the cilia axoneme marker acetylated tubulin (Acetyl-Tubulin), using anti-acetylated tubulin antibodies, and analyzed by immunofluorescence (IF) microscopy.

**Fig. 2:**
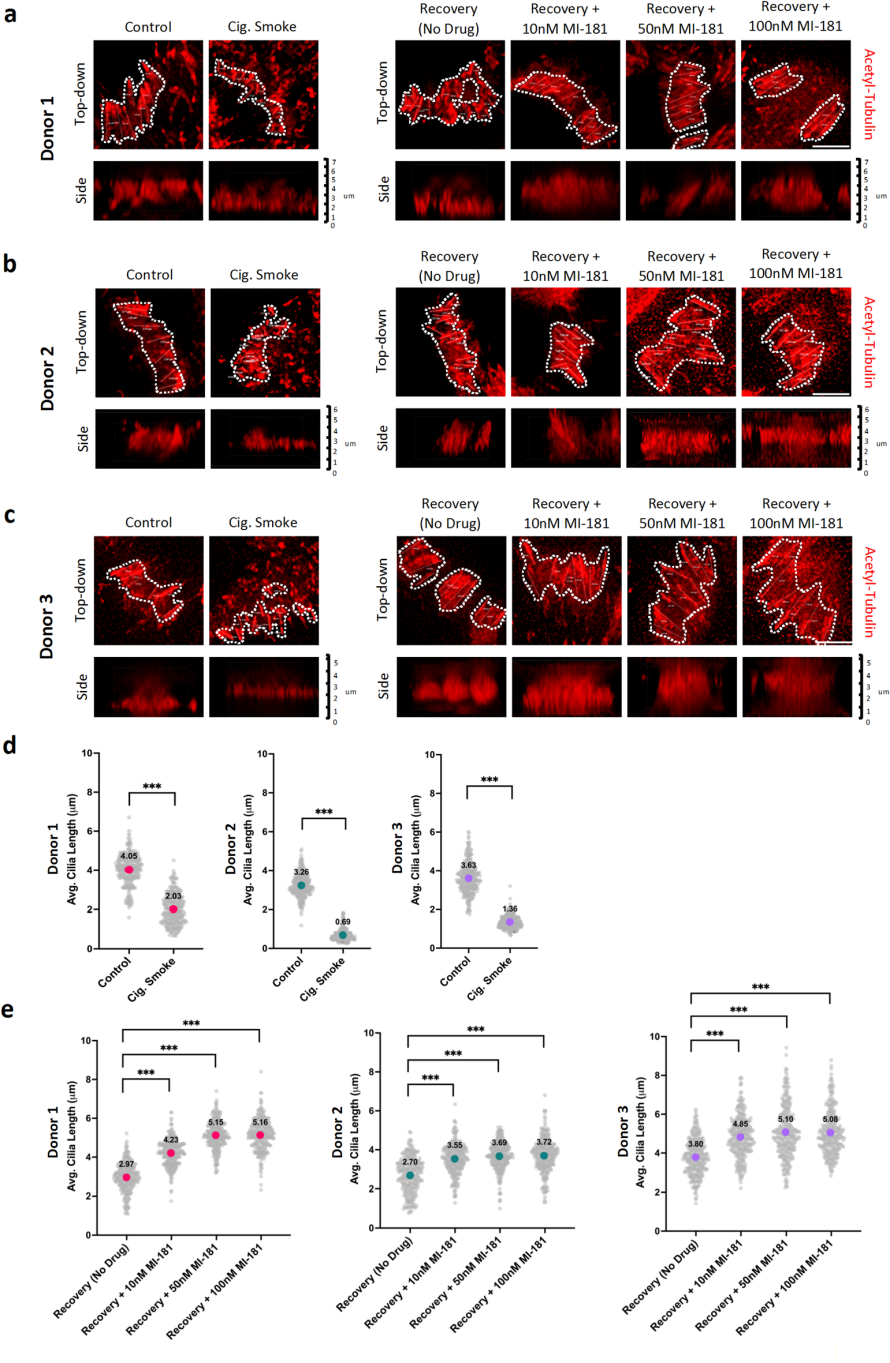
MI-181 enhances motile cilia length recovery of multiciliated cells in ABSC ALI cultures exposed to cigarette smoke. (**a-c**) IF-microscopy of three donor-derived ABSC ALI cultures. ABSC ALI cultures treated with or without cigarette smoke and allowed to recover in the absence or presence of increasing concentrations of MI-181 (10nM, 50nM, and 100nM). Two days post-recovery, ABSC ALI cultures were fixed and stained for cilia axonemes using an anti-acetylated tubulin antibody (red). Masks were placed on motile cilia patches of individual cells. Lower panels show the Z-stack side views of the cilia patches and their corresponding length measurements in micrometers (µm). Scale bars indicate 5 μm. (**d**,** e**) Graphs show a summary of the average cilia length (y-axis) for each treatment (x-axis) in each of the indicated donors. *n* = 300 individual cilia length measurements per condition per donor. Data are represented as the average ± standard deviation (SD). Asterisks indicate statistical significance as *** *p* < 0.001 compared to the controls. See the Quantification and Statistical Analyses section for details.

Visual inspection of the captured images showed that cigarette smoke treatment led to the presence of shorter cilia in all three donors (donors 1, 2, 3), and donors 2 and 3 showed a decrease in the density of cells that were ciliated, and MI-181-treatment appeared to restore cilia size (Fig. 1b-d). To quantify the effect of cigarette smoke and MI-181 on cilia length, we placed masks around cilia clusters and measured the lengths of 300 individual cilia per condition for each donor (Fig. 2a-c). This analysis revealed several insights. First, the average length of cilia in control untreated ABSC ALI cultures varied among the three donors (donor 1: 4.05 μm; donor 2: 3.26 μm; donor 3: 3.63 μm) (Fig. 2d). Second, cigarette smoke treatment led to a decrease in the length of cilia for all donors (donor 1: 2.03 μm; donor 2: 0.69 μm; donor 3: 1.36 μm) (Fig. 2d). Third, recovery from smoke-treatment allowed the cilia to increase in length (donor 1: 2.97 μm; donor 2: 2.70 μm; donor 3: 3.80 μm), and the addition of MI-181 at all concentrations (10nM, 50nM, and 100nM) enhanced the length of cilia during recovery in all donors (donor 1: range 4.23–5.16 μm; donor 2: range 3.55–3.72 μm; donor 3: range 4.85–5.08 μm), (Fig. 2e). Together, these results indicated that although cilia length can vary within donors, cigarette smoke treatment reduces cilia size, and MI-181 enhances cilia size during recovery.

**Fig. 3:**
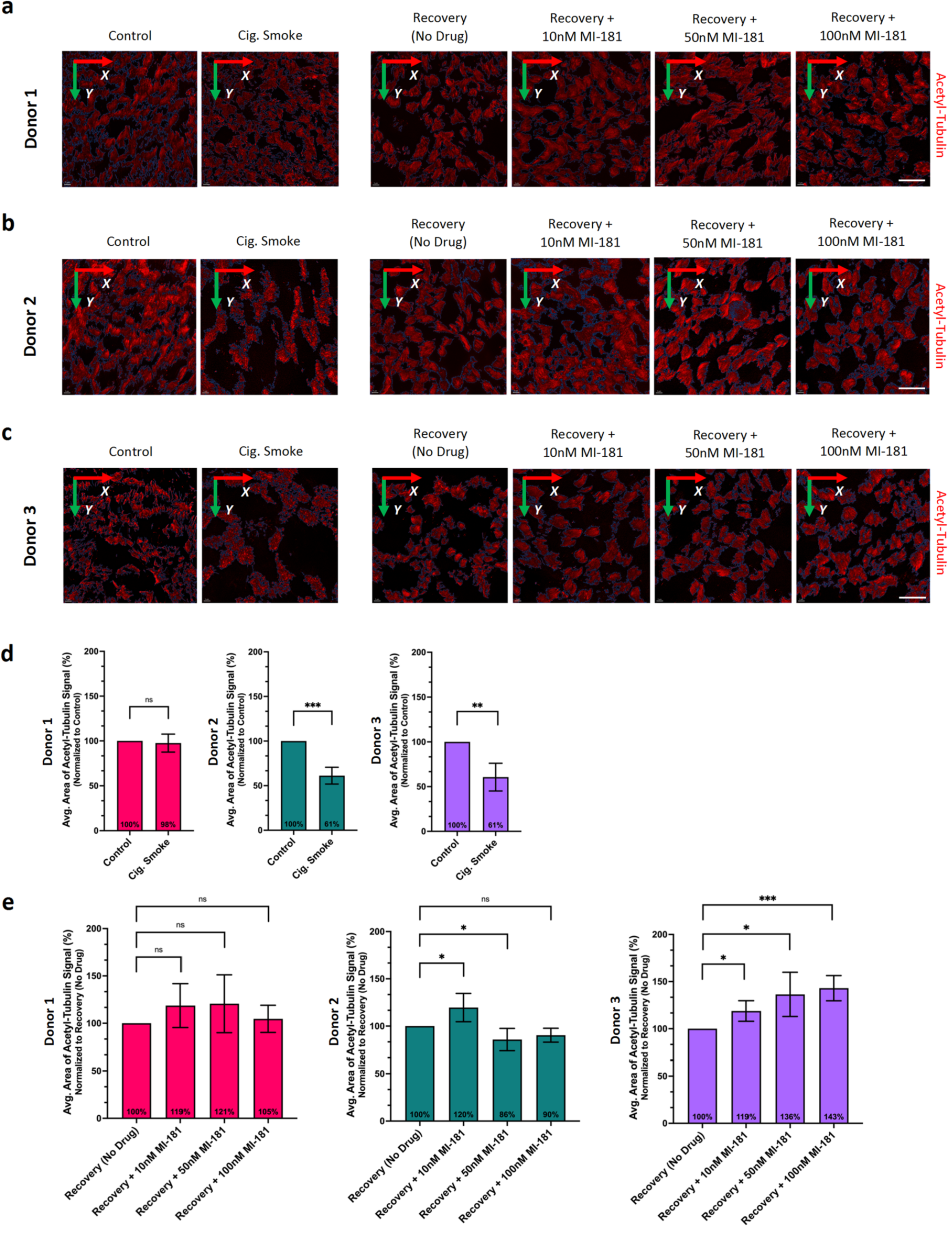
Analysis of MI-181’s effect on the area covered by motile cilia of multiciliated cells in ABSC ALI cultures exposed to cigarette smoke. (**a-c**) Same as in Fig. 1a-c, except that for each donor and condition, *n* = 5 100 μm x 100 μm images were analyzed for the average area covered by motile cilia (positive for anti-acetylated tubulin antibody, red) in ABSC ALI cultures. Scale bars indicate 20 μm. (**d**,** e**) Graphs show a summary of the average area covered by motile cilia (y-axis) for each treatment (x-axis), for each of the indicated donors. Data are represented as the average ± SD. Asterisks indicate statistical significance as * *p* < 0.05, ** *p* < 0.01, and *** *p* < 0.001 compared to the controls. See the Quantification and Statistical Analyses section for details.

Next, we sought to quantify the effect of cigarette smoke and MI-181 on the density of multiciliated cells within ABSC ALI cultures. To do this, Z-stacks of 100 μm x 100 μm images of ALI cultures from each donor and condition that had been stained for acetylated tubulin were analyzed using Leica Aivia artificial intelligence (AI) image analysis software to generate 3D images of the cilia on multiciliated cells (Fig. 3a-c). The average area covered by cilia patches was then quantified for each treatment condition per donor. This analysis revealed that for donor 1, cigarette smoke treatment had no effect on the average area covered by cilia (Fig. 3d). However, donors 2 and 3 both showed a 39% decrease in the average area covered by cilia (Fig. 3d). Similarly, for donor 1, MI-181 treatment did not show a significant effect on the average area covered by cilia during recovery from smoke (Fig. 3e). For donor 2, there was a significant increase in the average area covered by cilia in the 10nM MI-181-treated sample but not in samples treated with higher concentrations of MI-181 (Fig. 3e). For donor 3, MI-181 treatment led to a significant increase in the average area covered by cilia at all three concentrations (Fig. 3e). These results indicated that although the length of cilia was reduced upon smoke treatment in all three donors, only donors 2 and 3 showed a decrease in the number of multiciliated cells. Additionally, in donors in which smoke treatment reduced the average area covered by cilia (donors 2 and 3), 10nM MI-181 induced an increase in the area covered by cilia during recovery, whereas a higher concentration of MI-181 increased the area covered by cilia only in donor 3. These results also highlight the inter-donor heterogeneity in the average area covered by cilia and its response to smoke and MI-181 treatment.

### Analysis of FOXJ1 expression in cigarette smoke and MI-181-treated ABSC ALI cultures

**Fig. 4:**
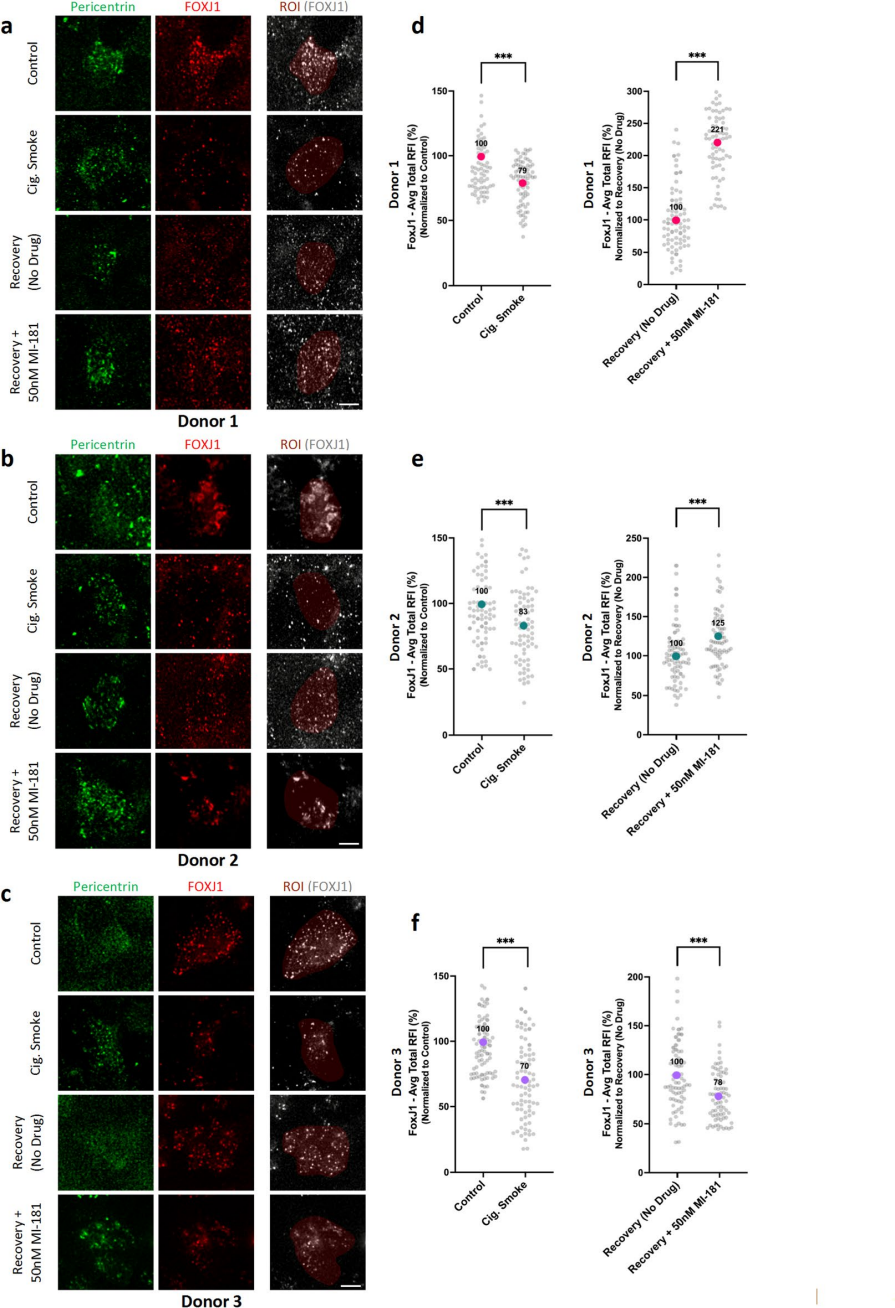
Analysis of MI-181’s effect on FOXJ1 levels in multiciliated cells of ABSC ALI cultures exposed to cigarette smoke. (**a-c**) Same as in Fig. 1a, except ABSC ALI cultures were fixed and co-stained for pericentrin (anti-pericentrin antibody, green) to visualize multiciliated cells and FOXJ1 (anti-FOXJ1, red) to visualize the levels of FOXJ1. The region of interest (ROI) mask used to quantify FOXJ1 levels are in the rightmost panels. Scale bars indicate 5 μm. (**d-f**) Graphs show a summary of the average total relative fluorescence intensity (RFI) for FOXJ1 (y-axis) for each treatment (x-axis), for each of the indicated donors. *n* = 75 cell images per condition per donor. Data are represented as the average ± SD. Asterisks indicate statistical significance as *** *p* < 0.001 compared to the controls. See the Quantification and Statistical Analyses section for details.

FOXJ1 is an important transcription factor crucial for the expression of numerous proteins that assemble motile cilia and the anchoring of basal bodies to template axonemal formation^20,21^. The levels of FOXJ1 often correlate with motile cilia formation, and cigarette smoke treatment can lead to decreased FOXJ1 levels in airway ALI cultures^12^. Therefore, we sought to measure the levels of FOXJ1 in cigarette smoke and MI-181-treated ABSC ALI cultures. Control (no smoke exposure) or cigarette smoke-exposed ALI cultures were allowed to recover in the presence or absence of DMSO control or 50nM MI-181 for two days (Fig. 4a-c). ALI cultures were then fixed with PFA and stained for pericentrin (using anti-pericentrin antibodies) to mark the base of motile cilia and distinguish multiciliated cells from monociliated cells, and FOXJ1 (using anti-FOXJ1 antibodies) (Fig. 4a-c). Aivia AI image analysis software was then used to generate a model capable of detecting multiciliated cells and measuring the average FOXJ1 total fluorescence intensity per cell (Fig. 4d-f). This analysis revealed that cigarette smoke treatment resulted in a decrease in FOXJ1 levels for all three donors (Fig. 4d-f). Additionally, MI-181 was able to enhance the levels of FOXJ1 during recovery for donors 1 and 2, but not for donor 3 (Fig. 4d-f). Donor 3 had a reduction in FOXJ1 levels in the MI-181 treated sample (Fig. 4f). These results indicated that FOXJ1 levels drop upon exposure to cigarette smoke and that MI-181 was able to enhance the levels of FOXJ1 during recovery from cigarette smoke in two out of three donors. These results also showed inter-donor heterogeneity in FOXJ1 levels in response to MI-181 treatment.

### MI-181-treated ABSC ALI cultures display normal motile cilia axoneme structure

**Fig. 5:**
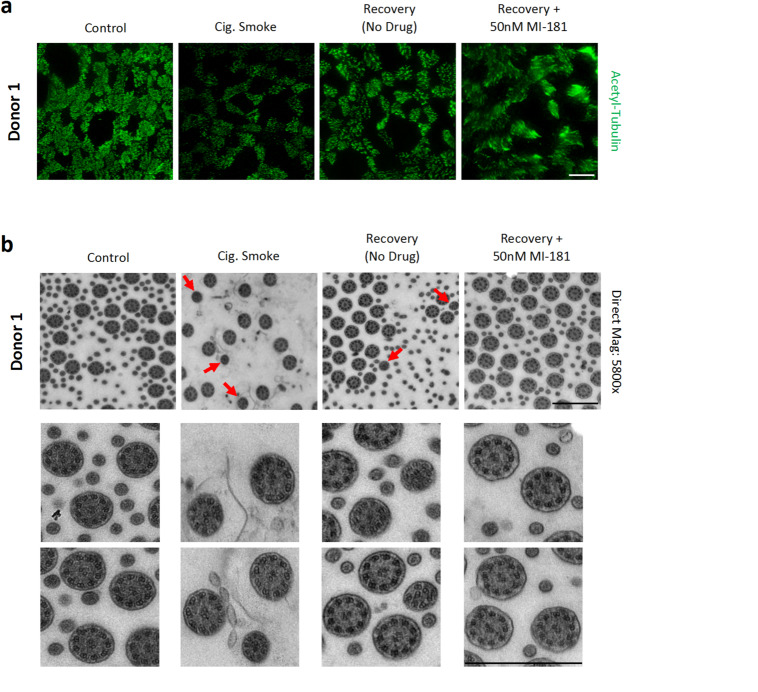
MI-181-treated motile cilia maintain a normal 9 + 2 arrangement of microtubules. (**a**,** b**) Same as in Fig. 1a, except that donor 1 ABSC ALI culture membranes were grown in parallel. One set of membranes were analyzed by IF microscopy to visualize motile cilia length and coverage area (**a**), and the other set was analyzed by transmission electron microscopy (TEM) (**b**). (**a**) IF-microscopy of donor 1-derived ABSC ALI cultures. The cultures were treated with or without cigarette smoke and allowed to recover in the absence or presence of 50nM MI-181. Two days post-recovery, ABSC ALI cultures were fixed and stained for cilia axonemes using an anti-acetylated tubulin antibody (green). Scale bar indicates 20 μm. (**b**) TEM analysis of 70 nm slices of flat-embedded donor 1-derived ABSC ALI cultures. Motile cilia axoneme cross sections of each condition are shown at 5,800X magnification. Lower panels show zoom views of two areas from each condition. Arrows indicate defective axoneme structures. Scale bars indicate 800 nm.

Prior studies demonstrated that the structure of motile cilia axonemes becomes compromised when ABSC ALI cultures are exposed to e-cigarette toxins^17^. Therefore, we sought to determine the effect of cigarette smoke and MI-181 treatments on the cilia axoneme structure of multiciliated cells in ABSC ALI cultures. To do this, duplicate sets of ABSC ALI culture membranes were established from donor 1 and exposed to no smoke (control) or to cigarette smoke and allowed to recover in the presence or absence of DMSO control or 50nM MI-181 for two days. One set of membranes was fixed, stained with anti-acetylated tubulin antibodies, and imaged by IF microscopy to follow the effect of cigarette smoke and MI-181 on motile cilia of multiciliated cells. Similar to our previous results (Figs. 1 and 2), cigarette smoke treatment resulted in a reduction in cilia length, whereas MI-181 treatment enhanced cilia length during recovery (Fig. 5a). The second set of membranes was fixed, flat-embedded in Eponate 12, and ultrathin sections of 70 nm thickness were prepared and analyzed by a transmission electron microscope (TEM). This analysis revealed several insights. First, cigarette smoke treatment led to an increase in motile cilia axoneme defects that deviated from the typical 9 + 2 arrangement of microtubules (Fig. 5b). These defects included changes in the orientation and arrangement of the microtubule doublets, as well as the separation of the central pair microtubules (Fig. 5b). Second, these axoneme microtubule arrangement defects persisted to a lesser degree in samples left to recover from cigarette smoke treatment for two days. Third, the structure of motile cilia that had been treated with 50nM MI-181 during recovery displayed a normal 9 + 2 arrangement of microtubules (Fig. 5b). Together, the TEM analysis of motile cilia on multiciliated cells showed that smoke treatment led to defects in the structure of motile cilia axonemes. Furthermore, MI-181-treated samples displayed normal microtubule axonemes.

## Discussion

The lack of effective therapeutics to treat airway diseases characterized by defective motile cilia remains a pervasive obstacle to long-term patient relief. Much is known about the protein components and signaling pathways crucial to the formation and maintenance of functional motile cilia. Additionally, considerable knowledge has accrued on how cigarette smoke and e-cigarette toxins lead to the dysregulation of these processes and the suppression of cilia formation and cilia length regulation, which inhibits the maintenance of healthy airways. However, fewer studies have been devoted to identifying and characterizing pharmacological leads to address these motile cilia defects and restore health to the airways. To address this, we sought to test the ability of the compound MI-181 to restore ciliation and cilia length to motile cilia of cigarette smoke-exposed ABSCs ALI cultures. Several insights have emerged from this study. First, donors can maintain variable lengths of motile cilia, and cigarette smoke induces the suppression of basal cilia length to varying degrees. Second, the effect of cigarette smoke on motile cilia formation also varies among donors. Third, in donor ABSC ALI cultures that displayed defects in ciliation and cilia length upon smoke treatment, 10nM MI-181 was able to enhance ciliation and cilia length during the recovery phase. Fourth, MI-181 treatment led to an increase in the levels of the motile cilia transcription factor FOXJ1 in ABSC ALI cultures from two of the three donors. Fifth, TEM analysis of ABSC ALI cultures treated with MI-181 revealed that motile cilia regained their normal structural integrity, exhibiting a 9 + 2 arrangement of microtubules. Together, these results suggest that MI-181 warrants further evaluation within the context of developing therapeutics for smoke-related airway diseases that are characterized by defective motile cilia.

The detailed mechanism by which MI-181 induces cilia growth remains incomplete. Using pharmacological agents to modulate the levels of soluble tubulin, Sharma et al.^22^, determined that the soluble levels of tubulin regulated primary cilia length. Given MI-181’s role in depolymerizing cytosolic microtubules^14^, we propose that the ability of MI-181 to induce cilia growth is through the generation of soluble tubulin that is available to build cilia. Although MI-181 treatment increased FOXJ1 levels during smoke recovery in two of the three donors, we believe this is a response to increased soluble tubulin levels rather than a direct effect of MI-181 on FOXJ1 stabilization. The FOXJ1 transcription factor is essential for building motile cilia through the expression of motile cilia-building factors, but is not essential for building primary cilia^23^, whereas the effect of MI-181 on cilia growth is seen in both motile (this study) and primary cilia^16^. A limitation of this study is the small number of donors used (3). While this is the first proof of concept study to demonstrate the potential of MI-181 in inducing motile cilia growth, an expansion in the number of donors could lead to defining the underlying mechanisms of the heterogeneity observed in response to MI-181 for some of the parameters measured, like the average ciliated area and the average levels of FOXJ1. Interestingly, our motile cilia TEM studies showed that circular microvilli structures of ~ 100 nm in diameter^24^ were also largely absent in cigarette smoke exposed ABSC ALI cultures (Fig. 5B). This observation is consistent with prior studies showing that cigarette smoke exposure leads to a decrease in the number or length of microvilli in airway epithelia^25,26^. However, the TEM analysis was conducted on samples from a single donor, and it would be necessary to perform this type of analysis across a large number of donors to probe a potential interplay between the number of healthy motile cilia and microvilli and MI-181’s effect on these structures. Although the TEM study showed that smoke exposed ABSC ALI cultures treated with MI-181 grew structurally normal cilia, future studies with functional readouts of cilia motility and mucociliary clearance will be necessary to fully establish MI-181 as a molecule that can not only restore cilia length and structure, but also function. Finally, our analysis on the effect of MI-181 on smoke exposed ABSC ALI culture motile cilia was conducted at a single short time point during smoke recovery, and it will be interesting to see if the longer exposure times to MI-181 would yield similar results.

## Methods

### Patient and sample collection

Tracheal and bronchial tissues were acquired from de-identified healthy human donors with no history of smoking, following lung transplantation at Ronald Reagan UCLA Medical Center and from the International Institute for the Advancement of Medicine (IIAM). All experimental protocols were approved by the Institutional Review Board-approved protocols at the David Geffen School of Medicine at UCLA, IRB exemption# 21–000390, and all methods were carried out in accordance with their relevant guidelines and regulations. All tissue was collected in a deidentified manner and informed consent was obtained from all subjects and/or their legal guardians. Airway basal stem cells (ABSCs) from three donors were used for all experiments.

### Airway basal stem cell isolation

Human ABSCs were isolated following our previously published methods^27,28^^29^. All steps were performed with the trachea in cold PBS with antimicrobials. Briefly, the airways were dissected, cleaned, and incubated in 50U/ml dispase for 30 min at room temperature. Tissues were then incubated in 10 mg/mL DNase for 30 min at room temperature. Epithelium was stripped and incubated in 0.25% Trypsin-EDTA for 30 min, shaking at 37 °C to generate a single cell suspension. Isolated cells were passed through a 40 μm strainer and plated for passaging and expansion. All cells used in these experiments were passage (P) 2–3 at the start of the ALI culture procedure.

### Air-liquid interface cultures

24-well 6.5 mm transwells with 0.4 μm pore polyester membrane inserts (Corning, Corning, NY, USA) were coated with 0.5 mg/mL collagen type IV from human placenta dissolved in acetic acid and diluted in cell-grade water at a ratio of 1:10. 100µL was added to each transwell, allowed to air-dry, and cross-linked with UV light for 30 min prior to cell seeding. ABSCs P2-3 were seeded at 80,000/transwell and grown in the submerged phase with PneumaCult-Ex Plus (StemCell Technologies, Cambridge, MA, USA) for 5 days with 500 µl in the basal chamber and 250 µl in the apical chamber. Confluent ABSCs were lifted to ALI day 0 and cultured with 500 µl PneumaCult-ALI (StemCell Technologies) in the basal chamber. ALIs are considered mature at ALI day 21. Cultures were exposed to cigarette smoke on ALI day 21 for three days and harvested for analysis at the indicated time points.

### Cigarette smoke and compound treatments

Primary human ALI cultures were placed without lids in a 3 L airtight chamber (Kent Scientific, Torrington, CT, USA) equipped with an inlet and outlet. An air pump (Adafruit Industries, New York, NY, USA), connected to the inlet and a research-grade cigarette 1R6F (University of Kentucky, Lexington, KY, USA) via an adaptor, was controlled by an Arduino UNO microcontroller with a relay shield (Seed Technology Co, Bellaire, TX, USA)^17^. To prevent vacuum sealing, one outlet remained open. Cigarettes were burned for two 3-second puffs. The chamber outlet was then sealed, and cells were incubated in smoke for 3 min before the chamber was opened to release residual smoke. This exposure protocol was repeated once daily for 3 consecutive days. After the final exposure, cells were either treated with MI-181 (Enamine, Kyiv, Ukraine) or left untreated, allowed to recover for 48 h, and then collected for downstream analysis.

### Immunofluorescence microscopy

Air-liquid interface membranes with cultured cells (treated or untreated) were removed and fixed with 100% methanol. The cells were rehydrated with PBT (PBS + 1% DMSO + 0.1% Triton X-100) and varying concentrations of methanol, i.e., with 75% methanol + 25% PBT for 5 min, followed by 50% methanol + 50% PBT for 5 min, and finally with 25% methanol + 75% PBT for 5 min. The fixed membranes were then washed with deionized water and permeabilized with ice-cold acetone for 7 min, followed by one wash with deionized water and three washes with PBT. The permeabilized membranes were blocked with PBT + 10% BSA for 2 h at room temperature before being incubated with 0.5 mg/mL Hoechst 33,342 and the indicated primary antibodies (prepared in PBT + 10% BSA) overnight at 4°C. Membranes were then washed with PBT four times (5 min each) and subsequently incubated with secondary antibodies (prepared in PBT + BSA) for 90 min at room temperature, followed by four washes with PBT. The cells were then clarified with varying concentrations of PBT and glycerol, i.e., with 75% PBT + 25% glycerol for 20 min, followed by 50% PBT + 50% glycerol for 20 min, and finally with 25% PBT + 75% glycerol for 20 min. The membranes were mounted with ProLong Gold Antifade mounting solution (Thermo Fisher Scientific, Waltham, MA, USA) on glass slides. Images were captured with a Leica MICA microscope (Leica Microsystems (Deerfield, IL, USA), 63x/1.40 NA oil objective, Leica Application Suite X software) and exported as.tiff files.

### Transmission electron microscopy

Air-liquid interface membranes with cultured donor 1 cells were fixed with 2% glutaraldehyde and 4% paraformaldehyde in 0.1 M sodium cacodylate buffer, pH 7.4, for 15 min at room temperature and then transferred to fresh fixative overnight at 4 °C. The membranes were then washed in 0.1 M sodium cacodylate buffer. After washing the membranes, they were transferred to glass vials. Samples were then post-fixed in 1% osmium tetroxide in 0.1 M sodium cacodylate buffer for 1 h and dehydrated through a graded series of ethanol concentrations. After infiltration with Eponate 12 resin (Ted Pella Inc., Redding, CA, USA), the sections were flat-embedded in fresh Eponate 12 resin and polymerized at 60 °C for 48 h. Ultrathin sections of 70 nm thickness were prepared and placed on formvar/carbon-coated copper grids (Ted Pella Inc.). Ultrathin sections were counterstained with uranyl acetate and lead citrate. The sections were examined using a JEOL 100CX transmission electron microscope at 60 kV, and images were captured by an AMT digital camera (Advanced Microscopy Techniques Corporation, model XR611) at the UCLA Brain Research Institute Electron Microscopy Core Facility. All chemicals were purchased from Electron Microscopy Sciences (Hatfield, PA, USA) unless otherwise noted.

### Antibodies

Antibodies used for immunofluorescence were anti-acetylated Tubulin (Cat#T6793 Sigma-Aldrich, Burlington, MA, USA, and Cat#ab179484 Abcam, Waltham, MA, USA), anti-FOXJ1 (Cat#14–9965-82 Thermo Fisher Scientific), and anti-Pericentrin (Cat#NB100-61071 Novus Biologicals, Centennial, CO, USA). Information on these antibodies and other key reagents used in this study is provided in Supplemental Table S1.

### Quantification and statistical analyses

For average cilia length measurements, cilia clusters in each condition and donor were outlined, and lengths (µm) were measured across the clusters. A total of 300 cilia measurements were taken for each treatment condition per donor using Leica LAS-X MICA software. Data graphs presented the average of 300 individual cilia lengths for each treatment condition per donor. For measuring the total area covered by cilia patches, five 100 μm x 100 μm images with appropriate exposures and z-stacks were selected and exported as.tiff files in Aivia. These images were then max projected, and the ‘Cell Count’ recipe was applied (Intensity threshold: 1500, Fill hole size: 0µm^2^, Smoothing factor: 1, Background removal: Skip, Object size: 1–100000µm^2^, and Subset filtering: None). Data graphs presented the average area covered by cilia patches in five 100 μm x 100 μm images for each treatment condition per donor. For measuring total FOXJ1 levels, images were exported as.tiff files in Aivia. These images were then max projected, and regions of interest (ROIs) were drawn around individual multi-ciliated cells. The ‘Cell Count’ recipe was applied to each ROI (Fill hole size: 100000px^2^, Background removal: 0, Contrast Threshold : 0, Smoothing factor: 4, Object size: 100–130000 px^2^ & Separation factor: 100). The total relative fluorescence intensity (RFI) for each ROI was then recorded which represented the total FOXJ1 intensity within each cell. Data graphs presented the average normalized RFIs in 75 cells for each treatment condition per donor. For all measurements, data were considered statistically significant when *P* < 0.05. Asterisks indicate statistical significance; * *P* < 0.05, ** *P* < 0.01, *** *P* < 0.001. All data graphs were generated with GraphPad Prism 10.

## Supplementary Information

Below is the link to the electronic supplementary material.

Supplementary Material 1

## Data Availability

The datasets used and/or analyzed during the current study are available from the corresponding author on reasonable request. The accompanying Supplemental Material includes Supplemental Table S1.
