# Supplementary material for: MI-181 enhances ciliation and cilia length in a cigarette smoke exposed airway epithelial model
Source: Sci Rep. 2026 Jan 24;16:6136. doi: 10.1038/s41598-026-37296-2 (PMC12901011; doi:10.1038/s41598-026-37296-2)
Supplement: Supplementary file 1 — Supplementary Material 1 [file 41598_2026_37296_MOESM1_ESM.pdf]

Table S1

REAGENT or RESOURCE	SOURCE	IDENTIFIER
**Antibodies**		
Mouse monoclonal anti-acetylated-Tubulin (clone 6-11B-1)	Sigma-Aldrich	Cat# T6793<br>RRID:AB_477585
Rabbit monoclonal Anti-alpha Tubulin (acetyl K40) antibody [EPR16772]	Abcam	Cat# ab179484<br>RRID: 2890906
Rabbit polyclonal anti-Pericentrin	Novus Biologicals	Cat# NB100-61071<br>RRID: AB_2283553
Mouse monoclonal anti-FoxJ1	Thermo Fisher Scientific	Cat# 14-9965-82<br>RRID: AB_1548835
Donkey polyclonal anti-Mouse IgG (H+L), Cy3 AffiniPure	Jackson ImmunoResearch Labs	Cat# 715-165-151<br>RRID: AB_2315777
Donkey polyclonal anti-Mouse IgG (H+L), Fluorescein (FITC) AffiniPure	Jackson ImmunoResearch Labs	Cat# 715-095-151<br>RRID: AB_2335588
Donkey polyclonal anti-Rabbit IgG (H+L), Fluorescein (FITC) AffiniPure	Jackson ImmunoResearch Labs	Cat# 711-095-152<br>RRID: AB_2315776
**Biological Samples**		
Human bronchiolar airway tissue specimens	Ronald Reagan UCLA Medical Center	N/A
**Cell Lines**		
Human primary airway basal stem cells	Gomperts' Laboratory	N/A
**Chemicals**		
ProLong Gold Antifade Mountant	Thermo Fisher Scientific	Cat# P36934<br>CAS: 56-81-5
Hoechst 33342	Thermo Fisher Scientific	Cat# H1399<br>CAS: 23491-52-3
DMSO	Corning	Cat# 25-950-CQC<br>CAS: 67-68-5
MI-181	Enamine	Cat# Z46018714<br>CAS: N/A
Trypsin-EDTA (0.25%)	Gibco	Cat# 25200072<br>CAS: 9002-07-7
Collagen from human placenta, Type IV	Sigma-Aldrich	Cat# C7521<br>CAS: 9007-34-5
PneumaCult-Ex Plus Medium	StemCell Technologies	Cat# 05040
PneumaCult-ALI Medium	StemCell Technologies	Cat# 05001
Glutaraldehyde Aqueous 8%, EM Grade	VWR	Cat# 100504-784<br>CAS: 111-30-8
Dispase	Thermo Fisher Scientific	Cat# NC9886504<br>CAS: 42613-33-2
Ethanol	Sigma	Cat# 459844<br>CAS: 64-17-5
Eponate 12 Embedding kit	Ted Pella	Cat# 18101

Sodium Cacodylate Buffer 0.4M	Electron Microscopy Sciences	Cat# 11655<br>CAS: 124-65-2
Propylene Oxide	Electron Microscopy Sciences	Cat# 20401<br>CAS: 75-56-9
Osmium Tetroxide	Electron Microscopy Sciences	Cat# 19150<br>CAS: 20816-12-0
Uranyl Acetate	Electron Microscopy Sciences	Cat# 22400<br>CAS: 541-09-3
Lead Citrate, Trihydrate	Electron Microscopy Sciences	Cat# 17800<br>CAS: 512-26-5
**Software and Algorithms**		
Leica Application Suite (LAS) X 6.2.2.28360	Leica Microsystems	https://www.leica-microsystems.com/
AIVIA 14.1.0	Leica Microsystems	https://www.leica-microsystems.com/
GraphPad Prism 10	GraphPad Software	https://www.graphpad.com/
Adobe Photoshop	Adobe	https://www.adobe.com/
BioRender	BioRender	RRID: SCR_018361
**Instrumentation/Other**		
Leica Mica Widefield Live Cell Microhub	Leica Microsystems	Cat# 630-3484
Inlet and Outlet Pump	Kent Scientific	N/A
Air Pump	Adafruit Industries	N/A
Arduino UNO Microcontroller	Seed Technology Co.	N/A
Certified Reference Cigarette 1R6F	University of Kentucky	2024-005CTRP
Formvar/Carbon coated copper slot grids	Ted Pella	Cat# 01805-F
24-well 6.5mm transwells with .4µm pore polyester membrane inserts	Corning	Cat# 3470
